# A few remarks on limits of research risks and research payments

**DOI:** 10.1007/s11019-022-10125-9

**Published:** 2022-11-09

**Authors:** Joanna Różyńska

**Affiliations:** Center for Bioethics and Biolaw, Faculty of Philosophy, University of Warsaw, Krakowskie Przedmiescie 3, 00-047 Warsaw, Poland

I would like to thank Greene and Brown (2022) for their comments on my paper “The ethical anatomy of payment for research participants” (Różyńska 2022), and for giving me an opportunity to clarify my position on research payment’s proportionality to risks and on an upper celling of permissible research risks, as well as on the community involvement in decision-making processes regarding research payments and risks.

Firstly, I want to underline that my article was not intended to provide a comprehensive and elaborated defense of a risk-based model of research payment. The paper highlights the lack of consensus regarding this matter among research ethicists and policy-makers, and it only mentions three arguments supporting the claim that an “adequate remuneration for research subjects should be proportionate also to the level of risk involved in participation”, leaving a detailed analysis of these issues for a different occasion. It is nevertheless necessary to notice that three arguments presented in the article may be employed to support different approaches to taking research risks under consideration in the process of determining the adequate level of payment: from those assuming that the degree of risk associated with the study may or should be considered as one of factors in calculating payment; to those – unlikely to be accepted by wider research ethics community – that consider payment to be a benefit counterbalancing research risks (cf. Wertheimer 2013).

Secondly, while the article remains mute on the question of a maximal level of acceptable risk in research, I fully agree with the commentators’ opinion that “there may be an upper limit on risk for which no payment amount should be paid” (Greene and Brown 2022:…). Contrary to bioethicists, who argue that the imposition of a maximal risk threshold in research would constitute a form of unjustified paternalism (e.g. Bergkamp 2004; Rajczi 2004; Shaw 2014), I claim that limiting research risks is not only justified, but it is necessary to maintain public trust in research enterprise and to protect research participants from excessive risks. I have extensively defended this position elsewhere (Różyńska 2015, 2023; see also Miller and Joffe 2009; Resnik 2012; Paquette and Shah 2020). One of my main arguments for setting a risk ceiling is that research volunteers are always the weaker party of the research practice. This is due to an inherent inequity in power between them and researchers (sponsors), which stems from three asymmetries: asymmetry in allocation and control over information regarding the study protocol (including information on involved risks); asymmetry in allocation and control over important risk-affecting aspects of the research (its design, conduct, and management); and asymmetry in the socioeconomic position of the parties. Due to these asymmetries, participants’ consent does not constitute a reliable and sufficient safeguard against undue or unwanted risks. Therefore, research participants need additional protection, among others, by the imposition of maximal level of permissible risk in research. Such a risk threshold should set a strict limit to any risk-based calculations of research payments.

Thirdly, I fully concur with the commentators’ view on the importance of involving community members in decision-making processes regarding the design, development, implementation, and monitoring of research, including the acceptability of associated risks and proposed research payments. This is entirely in a line with the CIOMS *International Ethical Guidelines for Health-related Research Involving Humans* (Council for International Organizations of Medical Sciences 2016), especially Guideline 7 (on community engagement), Guideline 4 (on potential individual benefits and risks of research), and Guideline 13 (on reimbursement and compensation for research participants). The document clearly states – what Greene and Brown also notice – that “an open and active process of community engagement is critical for building and maintaining trust among researchers, participants, and other members of the local community”, and that „failure to engage the community can compromise the social value of the research, as well as threaten the recruitment and retention of participants (Council for International Organizations of Medical Sciences 2016: 26, Commentary to Guideline 7). The CIOMS Guidelines *expressis verbis* recommend consultation with the local community to judge the appropriateness of a proposed compensation for research participation (2016: 54, Commentary to Guideline 13).

Moreover, the value of community engagement is a foundation of the “ELS procedural approach” which I have developed to provide research ethics committees (RECs) with procedural recommendations for identifying an ethically, legally and socially acceptable upper limit of research risk, especially in high-risk non-beneficial studies involving volunteers (Różyńska 2023). The ELS procedure comprises of three complementary and interdependent phases of risk-benefit analysis and evaluation: (1) **E**thical appraisal phase, in which REC should comprehensively describe all research risks and potential benefits, and carefully assess whether the risks are necessary and proportional to the study benefits, and whether the former has been adequately minimized, and the latter - maximized. (2) **L**egal appraisal phase, in which REC – usually with a support of an external legal advisor – should establish whether in a relevant legal system(s) the risks involved in the project fall within the scope of the *volenti non fit injuria* doctrine, i.e., whether a competent person may effectively consent for being exposed to a level of risk associated with the research. (3) **S**ocial appraisal phase, in which REC should ensure that representatives of relevant communities – especially the study population(s) and population(s) of potential research beneficiaries – consider risks involved in the study: (a) reasonable in relation to its potential benefits, (b) not too high *per se*, and (c) reasonable to take. “Risks are considered “reasonable to take”, if a significant number of representatives of relevant social groups, adequately informed about a project (particularly its goals, design, risks, benefits, recruitment strategies, and financial or other non-clinical gratifications for the participants) believes that the voluntary exposition to such risks can be a reasonable choice, rather than a decision made under undue inducement or other exploitative influences” (Różyńska 2023). Thus, the ELP approach is fully consistent with Greene and Brown’s comments on the importance of including community members in decisions regarding the appropriateness of research payments.

## Data Availability

Not applicable.
